# A Semi-Automated Technique Determining the Liver Standardized Uptake Value Reference for Tumor Delineation in FDG PET-CT

**DOI:** 10.1371/journal.pone.0105682

**Published:** 2014-08-27

**Authors:** Kenji Hirata, Kentaro Kobayashi, Koon-Pong Wong, Osamu Manabe, Andrew Surmak, Nagara Tamaki, Sung-Cheng Huang

**Affiliations:** 1 Department of Molecular and Medical Pharmacology, David Geffen School of Medicine at UCLA, University of California Los Angeles, Los Angeles, California, United States of America; 2 Department of Nuclear Medicine, Hokkaido University, Graduate School of Medicine, Sapporo, Japan; Banner Alzheimer's Institute, United States of America

## Abstract

**Background:**

^18^F-fluorodeoxyglucose (FDG) positron emission tomography (PET)-computed tomography (CT) has been an essential modality in oncology. We propose a semi-automated algorithm to objectively determine liver standardized uptake value (SUV), which is used as a threshold for tumor delineation.

**Methods:**

A large spherical volume of interest (VOI) was placed manually to roughly enclose the right lobe (RL) of the liver. For each voxel in this VOI, a coefficient of variation of voxel values (CVv) was calculated for neighboring voxels within a radius of *d/2*. The voxel with the minimum CVv was then selected, where a 30-mm spherical VOI was placed at that voxel in accordance with PERCIST criteria. Two nuclear medicine physicians independently defined 30-mm VOIs manually on 124 studies in 62 patients to generate the standard values, against which the results from the new method were compared.

**Results:**

The semi-automated method was successful in determining the liver SUV that was consistent between the two physicians in all the studies (*d* = 80 mm). The liver SUV threshold (mean +3 SD within 30-mm VOI) determined by the new semi-automated method (3.12±0.61) was not statistically different from those determined by the manual method (Physician-1: 3.14±0.58, Physician-2: 3.15±0.58). The semi-automated method produced tumor volumes that were not statistically different from those by experts' manual operation. Furthermore, the volume change in the two sequential studies had no statistical difference between semi-automated and manual methods.

**Conclusions:**

Our semi-automated method could define the liver SUV robustly as the threshold value used for tumor volume measurements according to PERCIST. The method could avoid possible subjective bias of manual liver VOI placement and is thus expected to improve clinical performance of volume-based parameters for prediction of cancer treatment response.

## Introduction

The clinical role of ^18^F-fluorodeoxyglucose (FDG) positron emission tomography (PET)-computed tomography (CT) in oncology has been well established [1]–[3]. For prediction of treatment response, maximal standardized uptake value (SUV_max_) has been used as the de facto standard for semi-quantitative measurement to assess the intensity of FDG uptake in the tumor [4]. Recently, an increasing number of investigators have been using volume-based parameters, such as metabolic tumor volume (MTV) and total lesion glycolysis (TLG) (TLG = SUV_mean_ times MTV) for lung cancer [5], [6], head-and-neck cancer [7], [8], gynecological cancer [9], [10], and many others. MTV is defined as the tumor volume within the boundary determined by some delineation method, such as fixed threshold (e.g. SUV≥2.5) [10]–[13], relative threshold (e.g. SUV≥40% of SUV_max_) [6]–[9], gradient-based [5], or region-growing method [14].

Wahl et al. proposed the criteria of PERCIST 1.0 in their comprehensive review paper on FDG PET-CT for prediction of treatment response, where volume-based parameters were recommended to be obtained with the use of liver SUV as a threshold to minimize the influence of inter-study variability of tumor SUV [4]. However, manual placement of liver VOI is subjective and could still give a biased threshold and thus a biased tumor volume measurement. According to Fencl et al., a 20% change in threshold led to 20% or more change in MTV [15]. In many cases, there are heterogeneous uptakes in the liver, even without liver diseases, which could lead to variability in the resulted thresholds. Thus, an automated method for objectively placing the liver VOI for FDG PET-CT without contrast enhancement is highly desirable.

A couple of such methods have been proposed recently. Bauer et al. proposed the use of a morphological technique to identify the liver [16]. The method is simple and fast. However, the method first converts the FDG SUV image to a binary image using a fixed threshold of SUV≥1. With the inter-study variability in SUV, a fixed SUV threshold is not expected to yield reliable results. The authors realized the problem, but no specific solution has been offered. Bi et al. proposed a more complicated method with the use of both CT and PET images to automatically identify the liver [17]. However, the processing time of their proposed method is too long (>2 hours) to be practical for routine use.

In this paper, we propose a new, simple, and semi-automated method that can determine the liver VOI quickly with no inter-operator variability. In addition, the method generates threshold values comparable to those established by experts using a manual procedure. This method is expected to reduce variability in tumor volume measurements and thus improve prediction of cancer treatment response.

## Methods

### The new algorithm

PERCIST 1.0 criteria recommend the use of a reference value calculated from a 30-mm spherical VOI in the right lobe (RL) of the liver. The new method was designed to automate the placement of this 30-mm VOI inside RL of the liver in a reproducible fashion.

Briefly, this algorithm searches the location where the coefficient of variation associated with a voxel (CVv) of the PET images is smallest. CVv is calculated by dividing the standard deviation (SD) by the mean within a sphere of a certain diameter. Since the liver is a large organ showing moderately high FDG uptake with higher homogeneity than neighboring FDG-avid organs (e.g., colon and kidney) in the absence of pathological conditions, CVv is expected to be lower for areas inside the liver where the corresponding VOI contains only liver tissue than for marginal areas containing both liver and adjacent tissues (e.g., fat, lung, and intestines).

**Figure 1:**
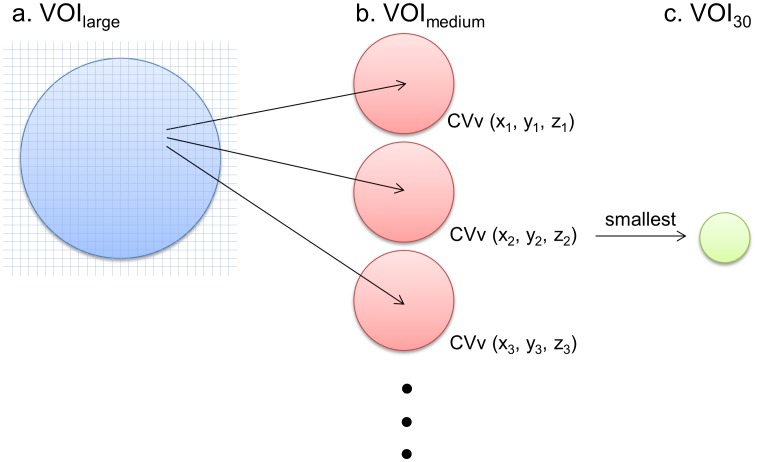
An illustration of the algorithm. (a) The spherical VOI_large_ confines the search area. (b) The spherical VOI_medium_ is defined for each pixel in VOI_large_ to calculate CVv ( =  standard deviation / mean). (c) VOI_30_ (sphere of 30-mm in diameter) is placed where CVv is smallest. The tumor delineation threshold is defined by the mean and SD within VOI_30_.

**Figure 2:**
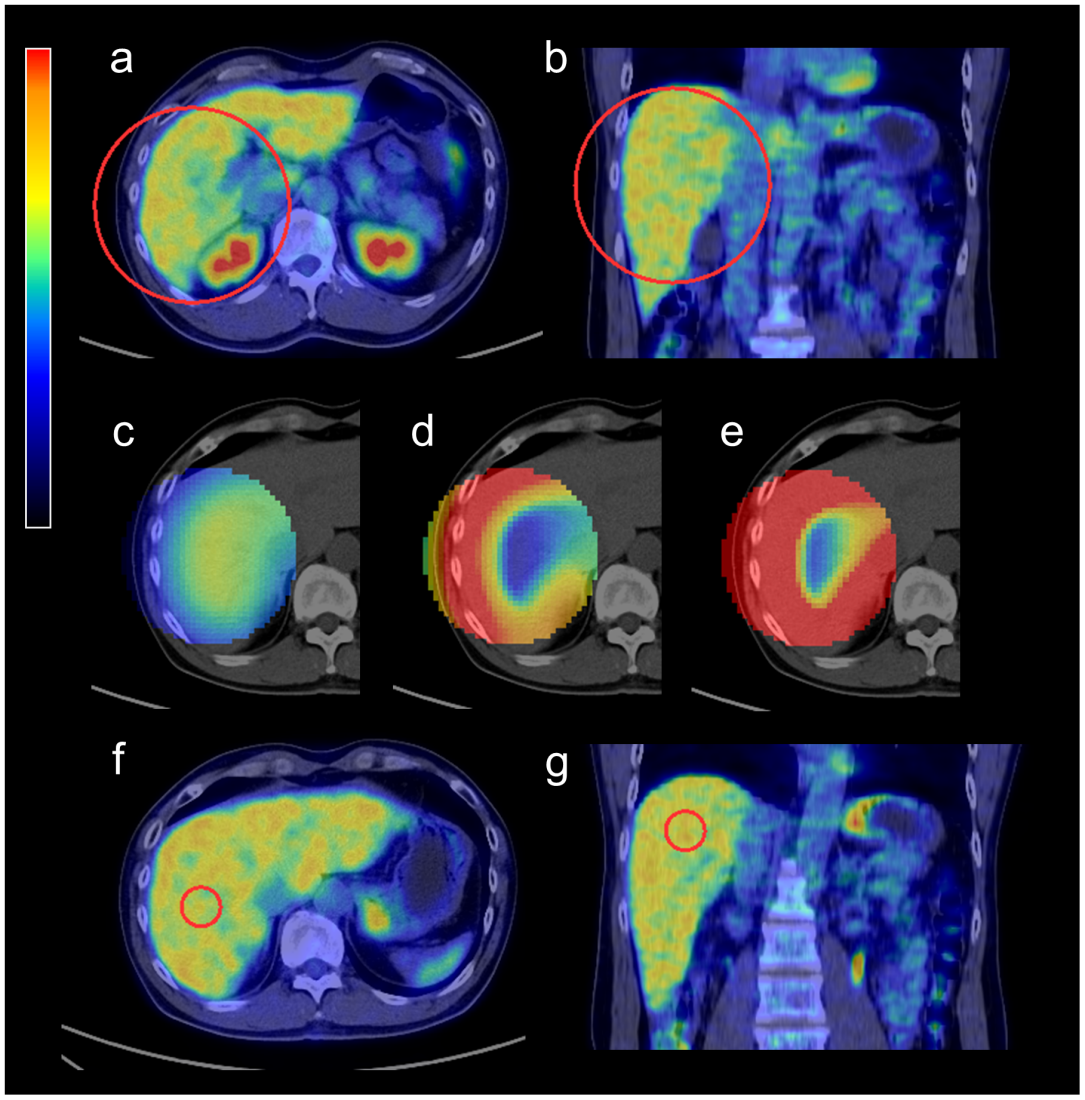
A representative case processed with the semi-automated algorithm. A 150-mm spherical VOI_large_ is manually placed to roughly enclose the right lobe of the liver (a, b). For each voxel within VOI_large_, a spherical VOI_medium_ (e.g., 80 mm in diameter) is defined, and mean (c), SD (d), and coefficient of variation of voxel (CVv) (e) within VOI_medium_ are calculated. A 30-mm VOI_30_ is placed where CVv is minimized (f, g). Image color scales are 0 to 4 SUV for (a–c, f, g), 0 to 1 SUV for (d), and 0 to 0.25 (unitless number) for (e).

Fig. 1 illustrates the proposed algorithm. First, a large spherical VOI (VOI_large_), which roughly encloses RL, is placed by an operator to confine a search area. Then, a medium-sized spherical VOI (VOI_medium_) is defined within each pixel of the search area (i.e., the VOI_large_) for calculation of CVv. After all CVv calculations, the voxel having the smallest CVv is identified, where a spherical VOI of 30 mm in diameter (VOI_30_) is placed (i.e., the center of the VOI_30_ is at that voxel location). The mean and SD within VOI_30_ is used for the following image processing as the threshold of tumor delineation as suggested by PERCIST 1.0. In our implementation, the size of VOI_large_ was fixed to 150 mm in diameter. We tested different sizes of VOI_medium_, including 40, 60, 80, 100, or 120 mm in diameter. A representative case is shown in Fig. 2.

The only human interaction required for this algorithm is to place VOI_large_. The program in which we implemented the algorithm is provided, along with its usage instructions, in a public website (www.metavol.org) for free user access.

### Study Subjects

Since the data were analyzed anonymously and retrospectively, no written or oral consent from each subject was obtained for the purpose of the current study. The Ethics Committee of Hokkaido University Hospital approved this retrospective study (#013-0259). A series of 1442 sequential studies of FDG PET-CT in Hokkaido University Hospital from July, 2012 to December, 2012 was reviewed. Ninety-six patients who underwent 2 or more exams were identified in this series. Thirty-one patients had 3 or more studies; in such cases, only the first two studies were included. Exclusion criteria are as follows: 1) fasting blood glucose level not measured or higher than 150 mg/dL, 2) known diabetes mellitus, 3) three or more metastatic lesions in the liver, as it becomes impossible to use liver uptake as background, 4) liver cirrhosis, 5) post-operative status of right lobectomy of the liver, and 6) significant technical error such as motion artifact and incomplete acquisition. Finally, 124 studies of 62 patients (age, 55±16 years old; body weight, 60±13 kg) were included, consisting of 24 patients with malignant lymphoma, 10 patients with head-and-neck cancer, 9 patients with skin cancers, and others. Every study was a part of patient care and performed because the patient's physician(s) considered FDG PET-CT to be necessary at that time to evaluate the patient for some clinical reasons such as staging, re-stating, and treatment response. The time interval between the two sequential examinations was 86.7±33.7 (range, 28–152) days.

### Image acquisition

All clinical PET-CT studies were performed with a Biograph 64 PET-CT scanner (Asahi-Siemens Medical Technologies Ltd., Tokyo, Japan). All the patients fasted for at least 6 hours before the injection of FDG (3.7 MBq/kg), and the emission scanning was initiated 60 minutes post injection. The actual time period from FDG injection to initiation of scanning was 58.3±7.1 minutes (mean±SD). For 110 out of 124 studies (89%), the uptake time was between 50 and 70 minutes, which satisfies the PERCIST's recommendation. The transaxial and axial field of views were 58.5 cm and 21.6 cm, respectively. A 3-minute emission scanning in 3-D mode was performed for each bed position. Attenuation was corrected with X-CT images acquired without contrast media. Images were reconstructed with an iterative method integrated with point spread function (TrueX) [18]. The reconstructed image had a spatial resolution of 8.4 mm FWHM and a matrix size of 168×168 with voxel size being 4.1×4.1×2.0 mm.

### Image Analysis

#### VOI placement

For each study, two experienced nuclear medicine physicians manually placed the 150-mm spherical VOI_large_ to roughly enclose the RL. As a control, the physicians also manually placed the 30-mm spherical VOI in RL at three different levels including upper (above the portal vein), middle (at the level of the right portal vein), and lower (below the portal vein) levels. The physicians paid careful attention not to place VOI_30_ in the area with a focally high or low uptake area.

#### Visual assessment of semi-automated VOI_30_

The VOI_30_ determined by the algorithm for each study was visually categorized into either successful or failed results. The placement was considered successful when the VOI_30_ from the two physicians were located exactly in the same place inside the RL. Otherwise, the placement was considered as failure. That is, if either of the following two cases occurred, the placement was considered a “failure.”

1. VOI_30_'s were at different locations when the two physicians ran the algorithm.
2. VOI_30_ included non-RL region.

#### Inter-study same-subject reproducibility of VOI_30_ location

**Figure 3:**
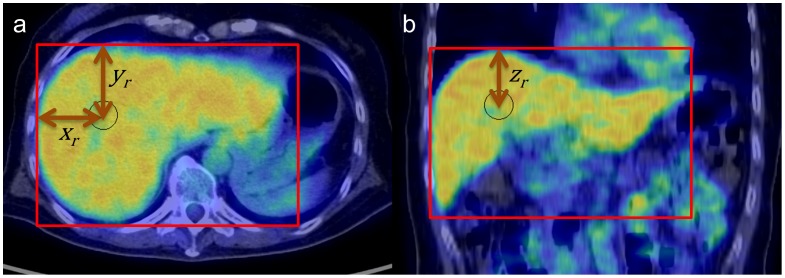
To evaluate inter-study same-subject reproducibility of the VOI_30_ location, a cuboid region (red rectangle) was manually created to precisely contain the whole liver. Location of VOI_30_ (black circle) was expressed as 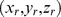 in the *liver* coordinate system.

Since the body location of the subject varied greatly for multiple studies, we used the following coordinate system within the liver to evaluate the inter-study reproducibility of VOI_30_ placement. The nuclear medicine physician (K.H.) defined a cuboid region that precisely circumscribed the whole liver (Fig. 3). The relative location of VOI_30_ in the liver R(x_r_, y_r_, z_r_) was calculated as follows:
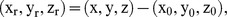
where (x_0_, y_0_, z_0_) represents the VOI_30_ location in the *whole-body* coordinate, and (x_0_, y_0_, z_0_) represents the *whole-body* coordinate values of the right, anterior, superior corner of the circumscribing cuboid. Thus, R(x_r_, y_r_, z_r_) represents the VOI_30_ location in the *liver* coordinate. Each patient underwent two PET-CT studies and had two *liver*-coordinate locations, R_1_ and R_2_. The Euclidean distance between R_1_ and R_2_ was then calculated.

#### Comparison of MTV

Before measuring MTV, the two physicians reached an agreement for each study about which uptake masses are considered as tumors. The tumor uptake that was proximal to non-specific uptake was not included because tumor boundary must be determined manually for these lesions, which is subjective and beyond the purpose of this study. After that, we applied mean +3 SD derived from VOI_30_ as the threshold. For all the pre-determined tumors, every voxel showing higher values than this threshold was considered as tumor. MTV was calculated as the sum of tumor volume in the entire body. Relative change was also calculated for each patient as follows:
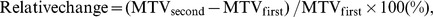
where MTV_first_ and MTV_second_ represent the MTV derived from first and second scans, respectively. MTV and relative change were compared between two physicians.

### Statistical analysis

Values are expressed as mean ±SD. The statistical software R (3.1.0) was used for all the statistical analyses. A paired t-test was used if the values could be considered as paired. The method of Holm was used to adjust the P-values for multiple comparisons [19]. Intra-class correlation (ICC) was used to evaluate inter-operator reproducibility [20]. The *psy* and *boot* packages for R were used to calculate ICC and its 95% confidential interval (CI) [21], [22]. P-values less than 0.05 were considered as significant.

## Results

### Manually placed VOI_30_

The mean SUV within the manually placed VOI_30_ by the first physician (P_1_) vs. the second physician (P_2_) from a total of 124 studies was shown in Table 1 (upper part). ICC between two physicians was highest for the upper level of the liver. Among a total of six manual values, only one combination reached significant difference (P_1_'s lower level vs. P_2_'s upper level VOI_30_'s, P<0.05).

**Table 1:** Mean SUV values within-VOI_30_ from different methods and physicians.

						Significant difference
		*n*	Physician-1	Physician-2	ICC (95% IC)	against manual methods
	VOI level					
Manual method	Upper	124	2.51±0.47	2.53±0.47	0.975 (0.961–0.985)	
	Middle	124	2.48±0.47	2.49±0.46	0.973 (0.946–0.984)	
	Lower	124	2.47±0.48	2.48±0.51	0.970 (0.949–0.983)	
	VOI_medium_ diameter					
Semi-automated method	40-mm	119	2.52±0.49		N/A	L1, L2, M1*
	60-mm	121	2.49±0.48		N/A	None
	80-mm	124	2.49±0.48		N/A	None
	100-mm	115	2.46±0.47		N/A	U1, U2*
	120-mm	71	2.38±0.48		N/A	U1, U2*

*L1: lower level by physician-1, M1: middle level by physician-1, U1: upper level by physician-1, U2: upper level by physician-2.

The threshold value, calculated as mean +3 SD within VOI_30_, was shown in Table 2 (upper part). Again, ICC was highest for upper level of the liver. No significant difference was observed among six values. The results for each patient are shown in Table S1.

**Table 2:** Mean ±3 SD values within-VOI_30_ from different methods and physicians.

						Significant difference
		*n*	Physician-1	Physician-2	ICC (95% IC)	against manual methods
	VOI level					
Manual method	Upper	124	3.14±0.58	3.15±0.58	0.971 (0.952–0.982)	
	Middle	124	3.11±0.60	3.11±0.58	0.970 (0.953–0.979)	
	Lower	124	3.13±0.64	3.11±0.62	0.939 (0.900–0.961)	
	VOI_medium_ diameter					
Semi-automated method	40-mm	119	3.02±0.59		N/A	U1, U2, M1, M2, L1, L2*
	60-mm	121	3.09±0.59		N/A	None
	80-mm	124	3.12±0.61		N/A	None
	100-mm	115	3.15±0.61		N/A	None
	120-mm	71	3.08±0.63		N/A	M1, M2*

*L1: lower level by physician-1, L2: lower level by physician-2, *M1: middle level by physician-1, M2: middle level by physician-2, U1: upper level by physician-1, U2: upper level by physician-2.

### VOI_30_ using the semi-automated method

#### Visual assessment of VOI_30_ placement

Table 3 summarizes the results of visual assessment of semi-automated VOI_30_. Using 80-mm VOI_medium_, VOI_30_ was successfully placed in the RL for all the studies. Using a 40-mm, 60-mm, 100-mm, or 120-mm VOI_medium_, the VOI_30_ failed to be placed appropriately in the RL in 5 (4.0%), 3 (2.4%), 9 (7.3%), and 53 (42.7%) studies, respectively. These findings suggested that the 80-mm VOI_medium_ was the best. The 120-mm VOI_medium_ was undesirable with the semi-automatic method. The computation time for 80-mm VOI_medium_ was 6.98±0.02 seconds for each study (Intel Core i7-3770 CPU at 3.40 GHz).

**Table 3:** Number of successful results by semi-automated method.

VOI_medium_ diameter (mm)	40	60	80	100	120
Successful *	119	121	124	115	71
	(96.0%)	(97.6%)	(100.0%)	(92.7%)	(57.3%)
Discrepant placement	0	0	0	0	7
Containing non-RL areas	5	3	0	9	46
Total	124	124	124	124	124

*The algorithm was considered *Successful* when the VOI_30_'s from the two physicians were located exactly in the same place inside the RL.

#### Inter-study same-subject reproducibility of VOI_30_ location

**Figure 4:**
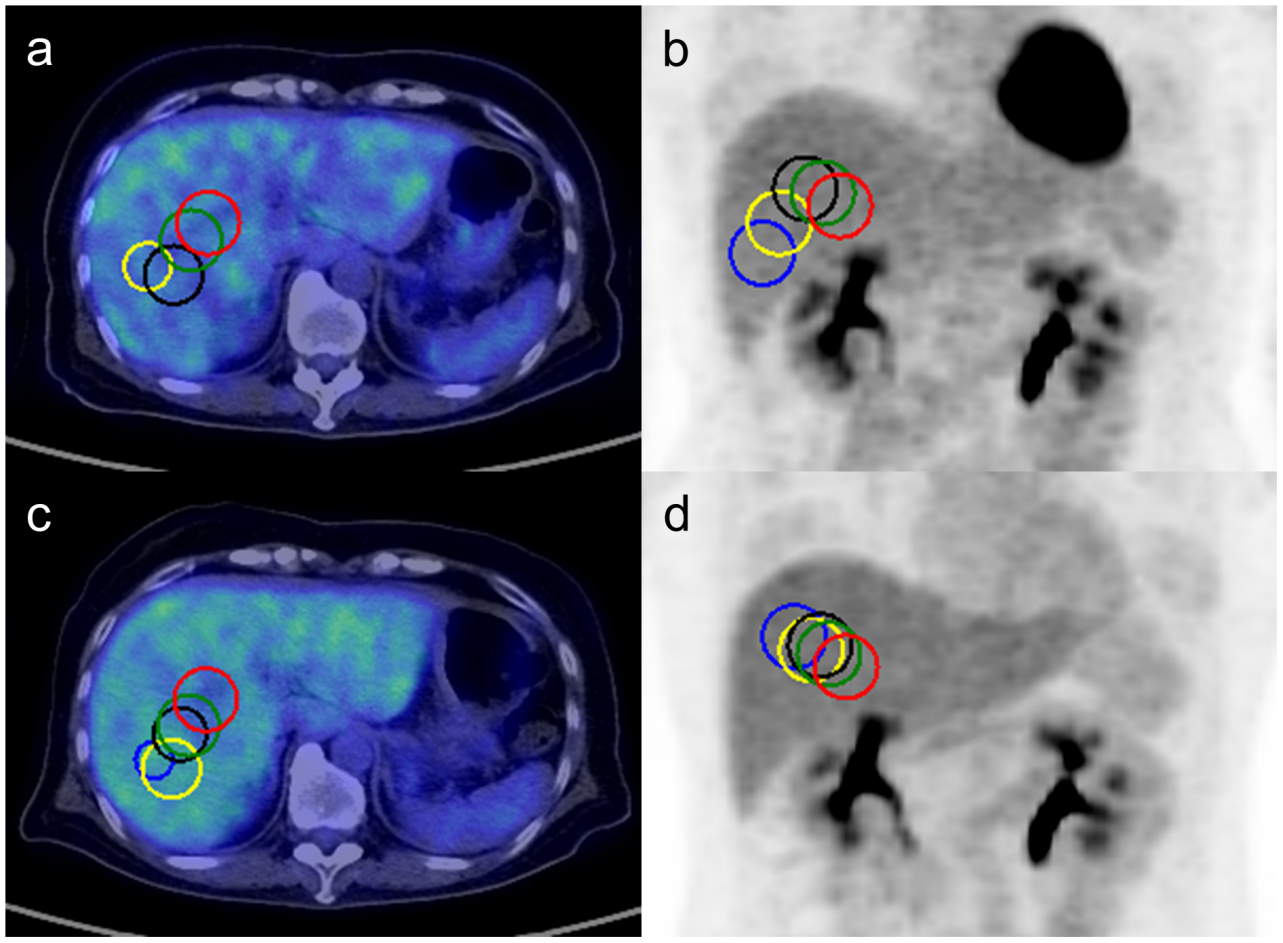
A representative case of two studies from the same patient. (a, b) first study and (c, d) second study. The VOI_30_'s defined using different size of VOI_medium_ (blue: 40 mm, yellow: 60 mm, black: 80 mm, green: 100 mm, red: 120 mm) are drawn on transaxial slices (a, c) and maximum intensity projection images (b, d). The smaller VOI_medium_ located VOI_30_ further from hepatic portal region with a larger distance between the first and second studies of the same patient than larger VOI_medium_'s did.

**Figure 5:**
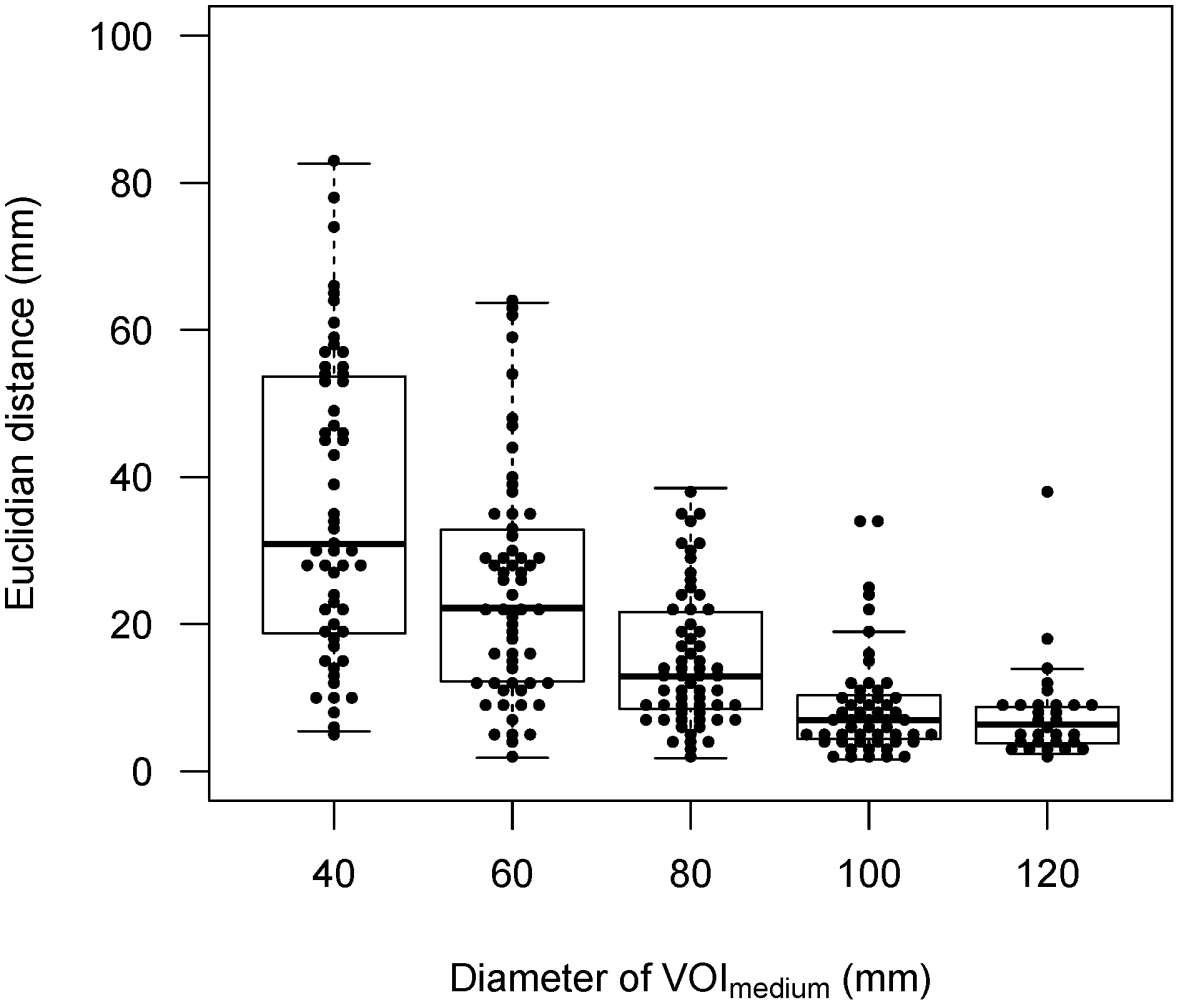
Euclidian distance between VOI_30_'s from two subsequent studies of the same patient by different VOI_medium_ sizes. Except the combination of 100-mm and 120-mm, all the combinations showed significant difference (P<0.001) after Holm's correction for multiple comparisons.

Fig. 4 shows an example of two studies from the same patient. The automated VOI_30_ using different sizes of VOI_medium_ was superimposed on PET-CT fused images and maximum intensity projection images. The small VOI_medium_ tended to locate VOI_30_ in peripheral areas with large distances between the first and second studies of the same patient. Fig. 5 shows the Euclidian distance between VOI_30_'s from two studies of the same patient. This analysis included only the VOI_30_ which were considered as successful at visual assessment. As the larger VOI_medium_ was used, the distance became smaller. All the combinations reached significant difference (P<0.001) except the combination of the 100-mm and 120-mm VOI_medium_'s. Inter-study reproducibility was thus considered to be highest with the 100-mm and 120-mm VOI_medium_, which was followed by the 80-mm VOI_medium_.

### Comparison of VOI_30_ values between manual vs. semi-automated methods

Tables 1 and 2 also summarize the mean of VOI_30_ (Table1, lower part) and mean +3 SD derived from VOI_30_ (Table 2, lower part), respectively. In the case that a 60-mm or 80-mm VOI_medium_ was selected, mean value of semi-automated VOI_30_ did not show significant differences from any manually derived VOI_30_. Conversely, selection of a 40-mm, 100-mm, or 120-mm VOI_medium_ resulted in significant differences for the within-VOI_30_ mean from some manually derived VOI_30_'s. Similarly, as Table 2 shows, in the case of 60-mm, 80-mm, or 100-mm for VOI_medium_, threshold values from the semi-automated VOI_30_ did not show significant differences from any manually derived VOI_30_.

Overall, the 80-mm VOI_medium_ was considered to be most desirable for this new algorithm, based on the criteria of achieving (a) the best successful rate, (b) a high inter-study same-subject reproducibility of VOI_30_ location, and (c) VOI_30_ values not significantly different from those by the manual method. For the manual VOI_30_, according to our results and previous work from another group [23], the upper VOI was considered least variable between physicians. In order to simplify the analyses, we hereafter included it only into the analysis for (i) automated VOI_30_ using 80-mm VOI_medium_, and (ii) manual VOI_30_ placed at upper level.

### MTV and its relative change

**Figure 6:**
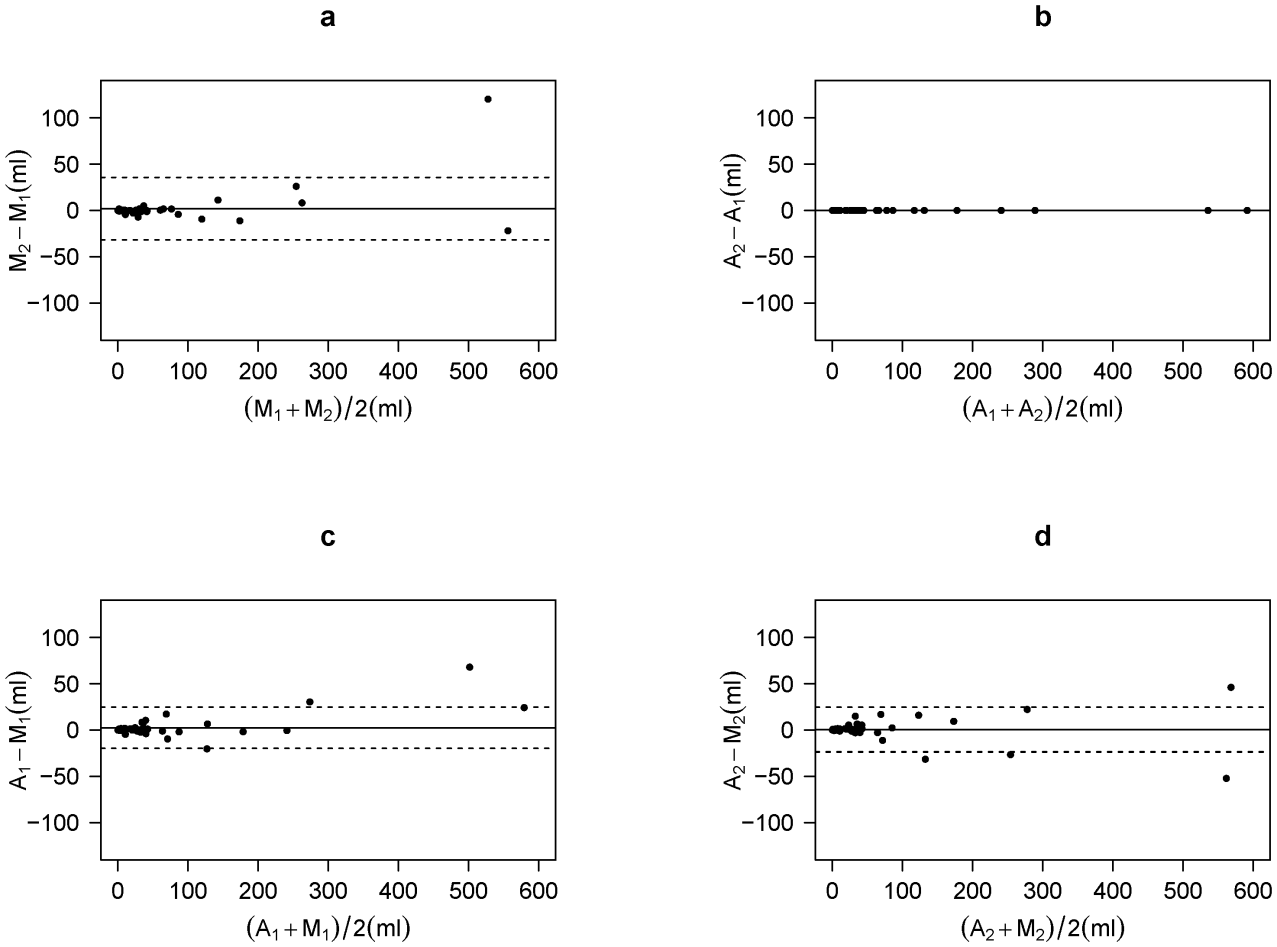
Bland-Altman plots of metabolic tumor volume. A_1_ and A_2_ represent value from the semi-automated method operated by physician-1 and -2, respectively. M_1_ and M_2_ represent manually derived value by physician-1 and -2, respectively. (a) M_1_ vs. M_2_, (b) A_1_ vs. A_2_, (c) A_1_ vs. M_1_, and (d) A_2_ vs. M_2_ are compared. Solid lines represent mean difference and dashed lines represent mean ±2SD.

A total of 57 out of 124 studies showed measurable tumors. MTV measured using the threshold from a manually derived VOI_30_ was 49.5±105.9 ml and 51.4±114.5 ml from P_1_ and P_2_, respectively. Fig. 6a shows Bland-Altman plot between two physicians. The ICC was 0.988 (95% CI: 0.972–0.999) with bias (P_2_–P_1_) being 1.95±16.9 ml. Paired t-test did not show a significant difference between the results by the two physicians. Using the automated algorithm, MTV was identical between the two physicians in all the patients (52.0±113.8 ml, Fig. 6b). No significant bias was found between the automated and manual methods (Fig. 6c, d).

**Figure 7:**
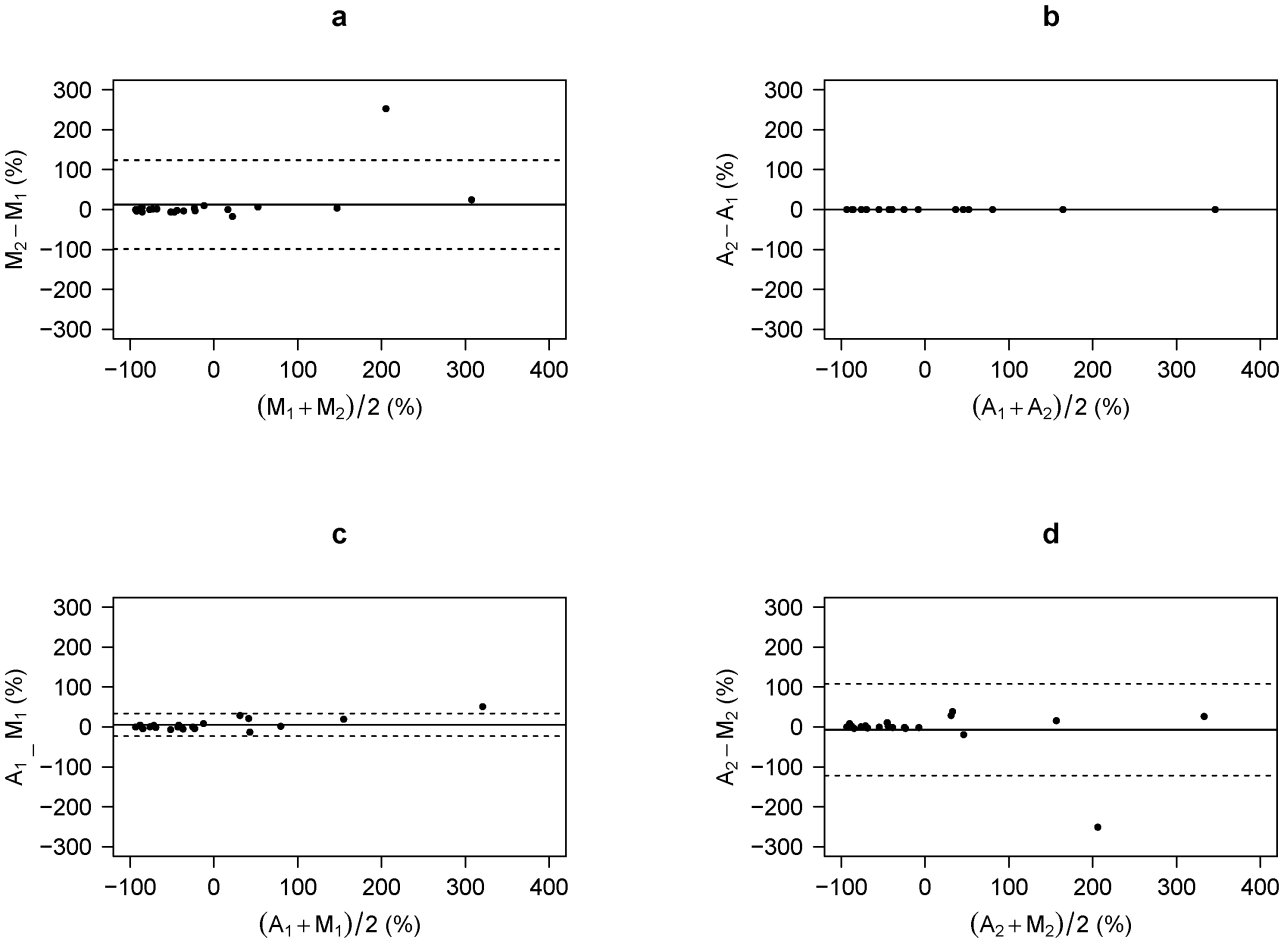
Bland-Altman plots of relative change of the metabolic tumor volume (MTV), calculated as [MTV_second_ – MTV_first_] / MTV_first_×100 (%). A_1_ and A_2_ represent value from the semi-automated method operated by physician-1 and -2, respectively. M_1_ and M_2_ represent manually derived value by physician-1 and -2, respectively. (a) M_1_ vs. M_2_, (b) A_1_ vs. A_2_, (c) A_1_ vs. M_1_, and (d) A_2_ vs. M_2_ are compared. Solid lines represent mean difference and dashed lines represent mean ±2SD.

A total of 21 out of 62 patients showing measurable tumors for both first and second studies were further analyzed. The relative changes of MTV based on manually derived VOI_30_ were −13.3%±94.5% and −0.9%±123.3%, respectively, for the two physicians (Fig. 7a). The ICC was 0.872 (95% CI: 0.603–0.997) with bias being 12.4%±55.6%. Paired t-test did not show a significant difference between the two physicians. Using the automated algorithm, the relative change was identical between the two physicians in all the patients (−7.9%±105.3%, Fig. 7b). No significant bias was found between automated and manual methods (Fig. 7c, d).

## Discussion

In this study, we tested a new semi-automated approach for VOI placement in RL. We applied this CV-based algorithm to clinical data and observed a high successful rate and high inter-operator reproducibility. In addition, the tumor volumes and the relative volume changes estimated using the semi-automated method were not significantly different from those obtained by experts using the manual method.

Although there are existing automated methods to identify the liver for FDG PET-CT [16], [17], they still need to be improved for clinical practice. On the other hand, our method is fast, simple to implement, reproducible, and highly successful (124/124 livers, 100%). Moreover, it generates equivalent values (threshold, tumor volume, and sequential volume change) to those manually determined by experts, indicating the high potential of the method in a clinical setting.

**Figure 8:**
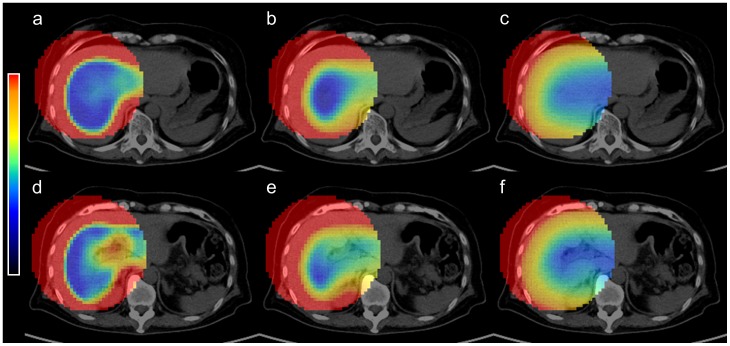
Example images of CVv by different VOI_medium_'s (a, d: 40 mm; b, e: 80 mm; c, f: 120 mm). When 40-mm VOI_medium_ was used (a, d), CVv seemed to be a non-convex function with many local minimums found in peripheral areas of the liver. When 80-mm VOI_medium_ was used (b, e), CVv seemed to be a convex function. The minimum voxel existed in the right lobe of the liver. When 120-mm VOI_medium_ was used (c, f), CVv seemed to be a convex function, but the minimum existed out of the liver. Image color scales are 0 to 0.25 for (a, d), 0 to 0.50 for (b, e), and 0 to 1.00 for (c, f).

Because CVv depends on the size of the VOI_medium_, we tried to identify the best VOI_medium_ using clinical data. First, based on successful rate with visual assessment, the 80-mm VOI_medium_ was the best with no failed results. For the other VOI_medium_'s (i.e., 40 mm, 60 mm, 100 mm, and 120 mm in diameter), the failed cases were mainly due to localization of VOI_30_ outside RL. Next, inter-study reproducibility was tested in distance analysis, where 100-mm and 120-mm VOI_medium_'s were the best followed by 80-mm VOI_medium_. When a smaller VOI_medium_ was used, the CVv seemed to be a non-convex function with many local minimums found in peripheral areas of the liver, which could explain the current results (Fig. 8). Another important requirement for an automated method is, in general, that the values from the automated method should not be significantly different from the values obtained by experts. When using 80-mm VOI_medium_, the calculated parameters (tumor volume and its relative change) had no significant difference from those determined by the manual method. All these test results support that the 80-mm VOI_medium_ should be used for this algorithm.

A recent research report showed that VOI_30_ placed in RL manually on the same FDG PET-CT study was reproducible between expert radiologists [23]. One may then argue that the automated method is not necessary. However, an expert needs to make every effort to avoid selecting focally high or low uptake areas in the liver as livers are not always homogeneous. Under the best conditions, there was still variability between operators. More importantly, one cannot rule out the possibility that an operator, intentionally or not, places the VOI_30_ in a relatively high / low uptake area in RL, yielding relatively high / low threshold value, and thus giving smaller / larger tumor volume. This might degrade the clinical value of MTV and TLG. We believe that objective methods should be used, especially for multi-center studies involving many investigators. We expect that our method will first be used in clinical trials and core. A wider experience gained in going through these uses should help determine the potential of the method for future routine clinical practice.

The 30 mm as the diameter of spherical VOI was suggested in PERCIST, but the reason for this is not very clear in the original publication. In practice, a 30-mm sphere is easy to be placed entirely within the right lobe of the liver for most patients. Although our method uses the 30-mm VOI in accordance with the suggested PERCIST guideline, the method does not require the diameter of the final VOI to be 30-mm. Therefore, the operation of the method will not be affected when future investigations determine an optimal VOI size.

PERCIST criteria have been widely used by many researchers since it was suggested in 2009 [24]–[29]. According to Skougaard et al., PERCIST has clear definitions and therefore more straightforward to use than conventional EORTC criteria [25]. However, even though there were studies comparing PERCIST with conventional criteria (e.g., EORTC) [25], [26], PERCIST has not been proven by prospective studies to be superior in terms of diagnostic accuracy. The readers should be aware of this point when using PERCIST in clinical studies.

Use of liver VOI as a tumor boundary threshold has a shortcoming that some tumors showing relatively low FDG uptake (e.g., hepatocellular carcinoma, renal cell carcinoma, and follicular lymphoma) could be overlooked. To measure the volume of such tumors, different thresholds or more sophisticated methods will be necessary. In addition, the true threshold to distinguish active tissues from necrotic tissues is dependent on many factors, such as tumor types, tumor shapes, and partial volume effects, in addition to liver SUV.

We need to mention four limitations regarding the current study. First, we did not test the cases in which tumor FDG uptake (e.g., uterine cervical cancer) was proximal to non-tumor FDG uptake (e.g., bladder) which necessitates human interaction to delineate the tumor. An automated method to solve this issue also should be developed. Second, we did not compare the tumor volume parameters with patient outcome, although a significant difference in this aspect between the semi-automated and manual methods is not expected because the two methods did not give significant differences in the total tumor volume (Fig. 6) and in the sequential change in tumor volume (Fig. 7).

As the third limitation, the liver SUV as a reference value is appropriate only when there is no evidence of hepatic metastasis or diffuse liver diseases such as cirrhosis, or prior resection of the right lobe. Therefore, the current method works only in such cases. In liver disease cases, PERCIST recommends use of VOI placed in the blood pool of the aortic arch as an alternative, although SUV in aortic arch has a larger variability than that in the liver [4]. A previous paper suggested a fully automated method of VOI placement in the aortic arch [16]. Such a method should be helpful to minimize inter-operator variability in these cases.

Finally, we tested the semi-automated method using the images generated from only a single PET-CT scanner (i.e., Biograph 64) and with a single reconstruction algorithm (i.e., TrueX). Since our method uses CVv in the images, the results may be influenced by the degree of image noise. In addition, the study population included only Asian people. There is a possibility that other patients from different populations have different sizes of the liver, which may necessitate a different VOI_large_ and VOI_medium_. Thus, this method needs to be evaluated further before more general uses such as in large-scale multi-center study.

## Conclusions

To overcome the shortcomings of previous automated methods, we proposed a new, simple, and semi-automated method that could place consistently the 30-mm VOI in the right lobe of the liver in accordance with PERCIST 1.0 criteria. The method achieved very high successful rates and high inter-operator reproducibility, and produced tumor volumes equivalent to those through the use of manually defined VOIs by nuclear medicine experts. Avoiding subjective bias, the semi-automated method will contribute to more reliable reference values for determining tumor boundaries and, consequently, is expected to provide better prediction of cancer treatment response and prognosis.

## Supporting Information

Table S1 **The results of volume of interest for each patient.**
(XLSX)
